# Metabolome alterations in severe critical illness and vitamin D status

**DOI:** 10.1186/s13054-017-1794-y

**Published:** 2017-07-28

**Authors:** Jessica Lasky-Su, Amber Dahlin, Augusto A. Litonjua, Angela J. Rogers, Michael J. McGeachie, Rebecca M. Baron, Lee Gazourian, Diana Barragan-Bradford, Laura E. Fredenburgh, Augustine M. K. Choi, Kris M. Mogensen, Sadeq A. Quraishi, Karin Amrein, Kenneth B. Christopher

**Affiliations:** 1 Channing Division of Network Medicine, Department of Medicine, Brigham and Women’s Hospital, Boston, MA USA; 2 Pulmonary and Critical Care Division, Channing Division of Network Medicine, Department of Medicine, Brigham and Women’s Hospital, Boston, MA USA; 3 Pulmonary & Critical Care Medicine, Stanford University Medical Center, Stanford, CA USA; 4 Pulmonary and Critical Care Division, Department of Medicine, Brigham and Women’s Hospital, Boston, MA USA; 5 Pulmonary and Critical Care Medicine, Lahey Hospital & Medical Center, Burlington, MA USA; 6 Department of Medicine, New York-Presbyterian Hospital, New York, NY USA; 7 Department of Nutrition, Brigham and Women’s Hospital, Boston, MA USA; 8 Department of Anesthesia, Critical Care and Pain Medicine, Massachusetts General Hospital, Boston, MA USA; 9 Division of Endocrinology and Metabolism, Department of Internal Medicine, Medical University of Graz, Graz, Austria; 10 Renal Division, Channing Division of Network Medicine, Department of Medicine, Brigham and Women’s Hospital, 75 Francis Street, MRB 418, Boston, MA 02115 USA

**Keywords:** Vitamin D, Metabolite, Metabolomics, Homeostasis, Critical illness

## Abstract

**Background:**

Metabolic homeostasis is substantially disrupted in critical illness. Given the pleiotropic effects of vitamin D, we hypothesized that metabolic profiles differ between critically ill patients relative to their vitamin D status.

**Methods:**

We performed a metabolomics study on biorepository samples collected from a single academic medical center on 65 adults with systemic inflammatory response syndrome or sepsis treated in a 20-bed medical ICU between 2008 and 2010. To identify key metabolites and metabolic pathways related to vitamin D status in critical illness, we first generated metabolomic data using gas and liquid chromatography mass spectroscopy. We followed this by partial least squares-discriminant analysis to identify individual metabolites that were significant. We then interrogated the entire metabolomics profile using metabolite set enrichment analysis to identify groups of metabolites and pathways that were differentiates of vitamin D status. Finally we performed logistic regression to construct a network model of chemical-protein target interactions important in vitamin D status.

**Results:**

Metabolomic profiles significantly differed in critically ill patients with 25(OH)D ≤ 15 ng/ml relative to those with levels >15 ng/ml. In particular, increased 1,5-anhydroglucitol, tryptophan betaine, and 3-hydroxyoctanoate as well as decreased 2-arachidonoyl-glycerophosphocholine and N-6-trimethyllysine were strong predictors of 25(OH)D >15 ng/ml. The combination of these five metabolites led to an area under the curve for discrimination for 25(OH)D > 15 ng/ml of 0.82 (95% CI 0.71–0.93). The metabolite pathways related to glutathione metabolism and glutamate metabolism are significantly enriched with regard to vitamin D status.

**Conclusion:**

Vitamin D status is associated with differential metabolic profiles during critical illness. Glutathione and glutamate pathway metabolism, which play principal roles in redox regulation and immunomodulation, respectively, were significantly altered with vitamin D status.

**Electronic supplementary material:**

The online version of this article (doi:10.1186/s13054-017-1794-y) contains supplementary material, which is available to authorized users.

## Background

Low vitamin D status is common in the intensive care unit (ICU) [1–3]. Several observational studies in critically ill cohorts suggest that vitamin D status is associated with important clinical outcomes [1–3]. In particular, low vitamin D status is associated with increased risk of sepsis and with worse outcomes in patients with sepsis [4, 5]. Moreover, recent studies support vitamin D as a potential therapeutic agent in hospitalized patients [6, 7].

Vitamin D has broad biological effects on nuclear transcription, cell cycle regulation, differentiation, and apoptosis [8]. Vitamin D metabolic enzymes and vitamin D receptors have a wide tissue distribution, reflecting the involvement of vitamin D in the metabolism and function of many cell types [9]. Indeed, differential metabolic profiles are demonstrated in ambulatory patients who respond to vitamin D supplementation relative to those who do not [10, 11]. Since metabolic homeostasis is often disrupted in critical illness, substantial alterations of several intrinsic pathways can be expected in septic patients [12]. Only a limited number of metabolomic studies have been published to date in experimental sepsis models [13], pediatric sepsis [14], and critically ill adults [15].

While some existing data support anti-inflammatory and immune modulating effects related to vitamin D supplementation [16], and while metabolomic approaches are used to understand the pleiotropic effects of Vitamin D [17, 18], there is limited understanding of the metabolic alterations associated with low vitamin D status in critical illness. Therefore, we analyzed metabolite profiles with regard to vitamin D status in a prospective study of adult patients with systemic inflammatory response syndrome (SIRS) and sepsis [19]. We hypothesized that the metabolomic profile of patients with severe critical illness near the time of ICU admission is influenced by vitamin D status and that this metabolic difference in turn can illuminate important biologic pathways that may contribute to pathogenesis and prognosis.

## Methods

### Study design and patients

The Registry of Critical Illness (RoCI) is a registry of adult medical ICU patients based at the Brigham and Women’s Hospital (Boston, MA, USA), created to record patient data and store samples for plasma, RNA/DNA analysis, and protein isolation. The protocol for patient recruitment has been previously described at length [19]. Between September 2008 and May 2010, 90 medical ICU patients had metabolic profiling: 29 of these patients satisfied SIRS criteria, 30 satisfied criteria for sepsis, and 31 satisfied criteria for sepsis and acute respiratory distress syndrome (ARDS) [20]. Patients were not selected with regard to risk of death or any known metabolic feature. We conducted a sub-analysis involving 65 RoCI patients who had been selected for metabolic profiling, and in whom plasma was available for measuring 25(OH)D levels (Additional file 1, Fig. 1).

**Fig. 1:**
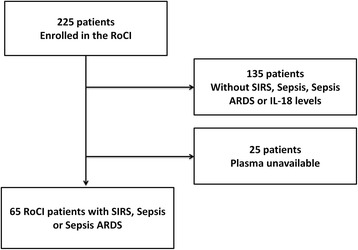
Flow chart of the cohort. *ARDS* acute respiratory distress syndrome, *SIRS* systemic inflammatory response syndrome, *RoCI* Registry of Critical Illness

Demographic and physiologic data were collected from the clinical record as described previously [19]. In addition to data collected by the RoCI, additional data on all patients were compiled through a well-described computerized registry, called the Research Patient Data Registry (RPDR) [21] as outlined in Additional file 1.

Plasma 25(OH)D level was measured using plasma samples from the same day as the plasma sample that was used for metabolic profiling. All 25(OH)D levels were measured via the competitive chemiluminescence immunoassay (CLIA) using the DiaSorin LIAISON 25-OH Vitamin D Total assay [22, 23]. Serum 25(OH)D levels were dichotomized a priori into low (≤15 ng/ml) and normal (>15 ng/ml) groups based on large studies performed by our group in the ICU under study, which consistently found differential outcomes at this cut point [2–4, 24].

Metabolomic profiling identified 411 metabolites for the complete RoCI cohort (N = 90 plasma samples within 72 hours of ICU admission) using Metabolon, Inc. [20]. Gas and liquid chromatography mass spectroscopy (GC-MS, LC-MS) were performed as described previously [25, 26]. We removed metabolites with the lowest IQR of variability in the RoCI data, leaving 308 metabolites. All metabolite concentrations were log2 transformed to normalize the data, which were utilized for all of the models and all of the metabolite data analyses. Details on metabolomic sample processing have been previously described at length [20].

**Fig. 2:**
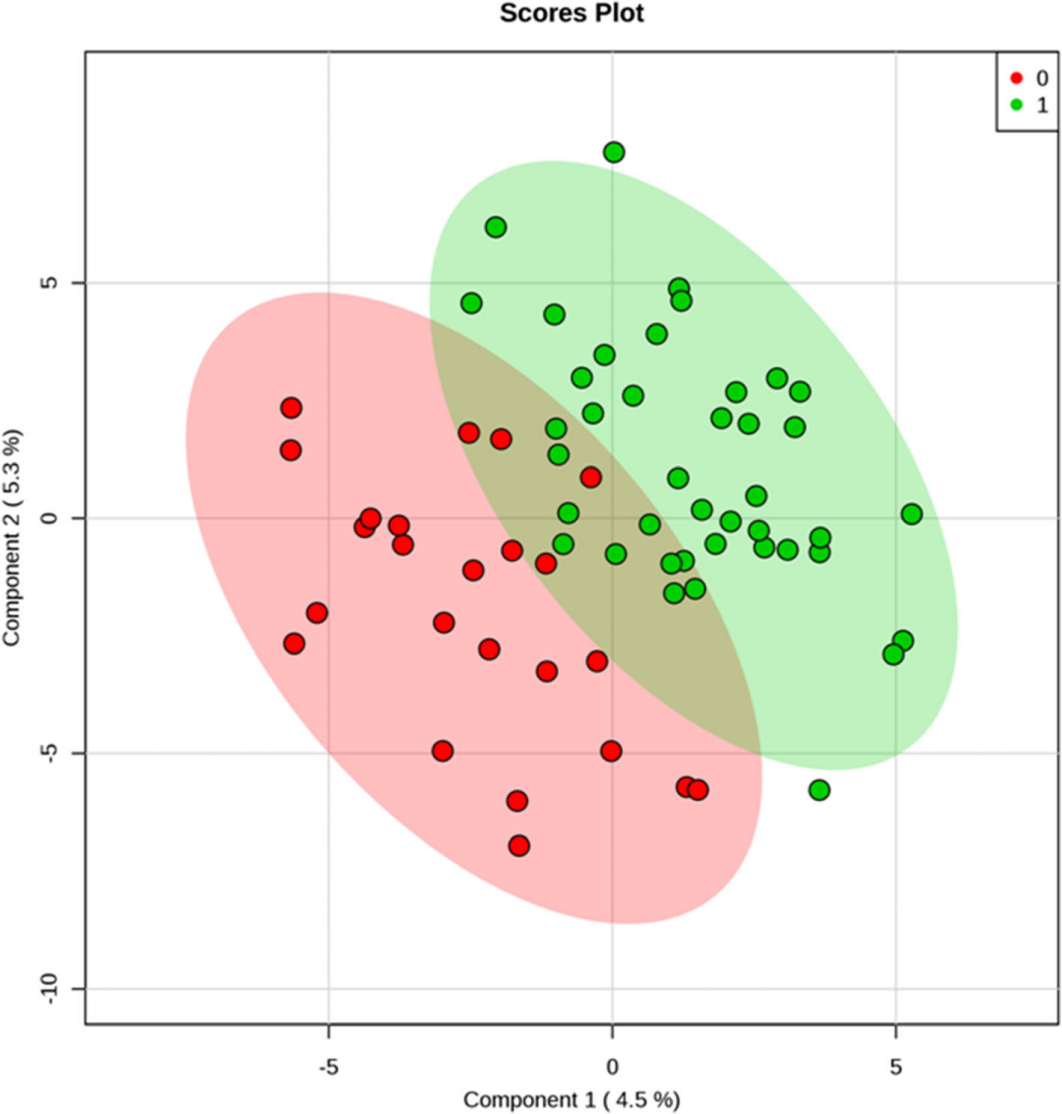
Partial least squares-discriminant analysis (PLS-DA). Cross-validated PLS-DA score plot for comparison of the global metabolite profiles of 24 patients with 25(OH)D ≤ 5 ng/ml (*red*) and 41 patients with 25(OH)D >15 ng/ml (*green*) shows the separation achieved according to vitamin D status. The *p* value based on permutation is 0.033 (66/2000). *Colored circles* represent 95% confidence intervals. *Colored dots* represent individual samples: *4.5%* and *5.3%* are the scores of *component 1* and *component 2*, respectively, in the PLS-DA analysis

**Fig. 3:**
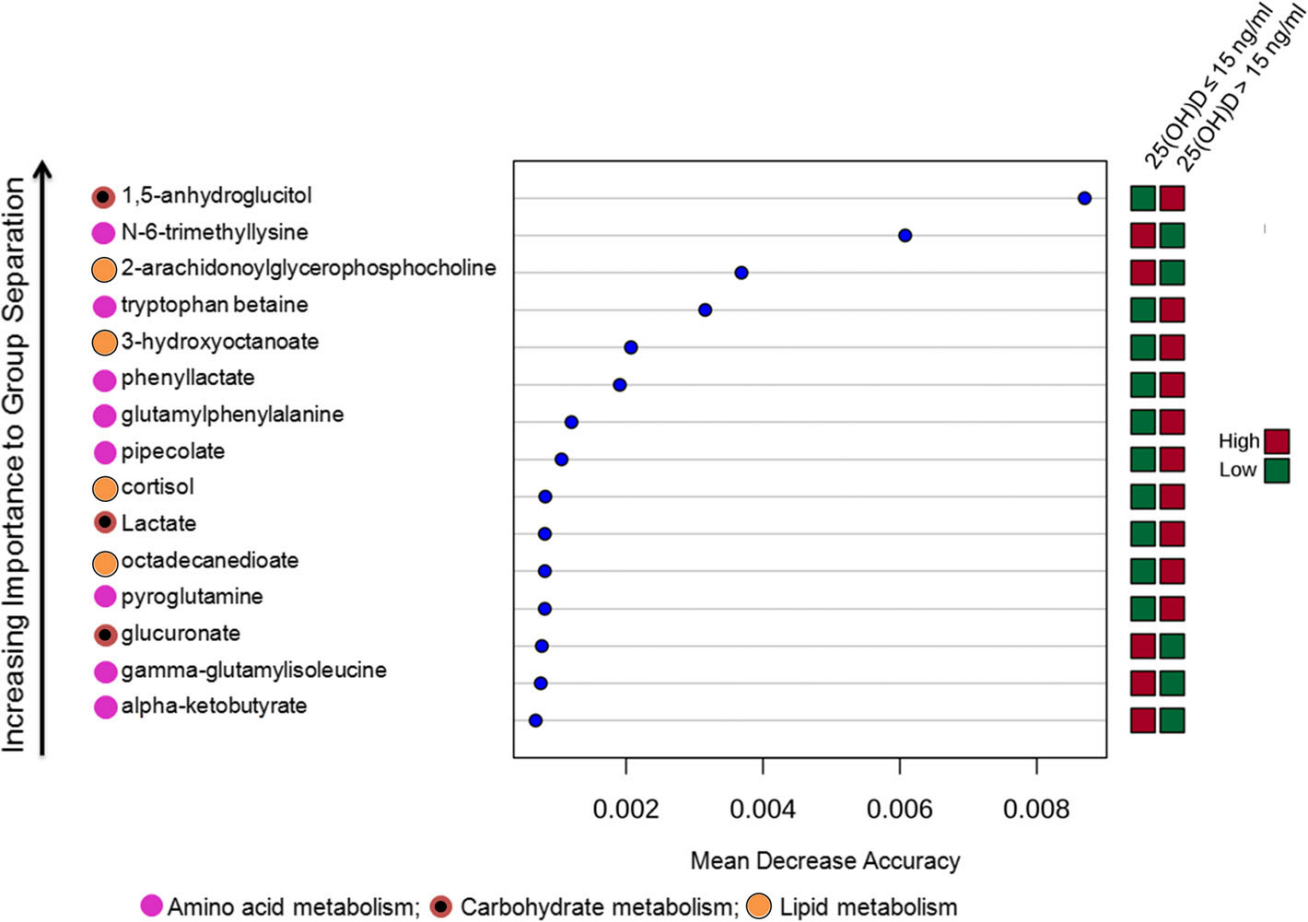
Vitamin D deficiency biomarker identification by global metabolomics. To relate vitamin D status to the blood metabolite data, we used random forest (RF) predictors. An RF importance measure was used to rank metabolites according to their prognostic importance for vitamin D status. The *colored boxes* on the *right* indicate the relative concentrations of the corresponding metabolite in the vitamin D groups. Metabolite classes (amino acid metabolic, carbohydrate metabolism, lipid metabolism) are indicated by the *colored circles*

We utilized MetaboAnalyst 3.0 software (www.metaboanalyst.ca) to identify key metabolism alterations related to vitamin D status [27]. We identified the group of metabolites that best discriminate between individuals with low and normal vitamin D status using partial least squares-discriminant analysis (PLS-DA) (Fig. 2) and identified the metabolites responsible for the overall discrimination ability (Fig. 3). PLS-DA model validation was determined by permutation tests based on separation distance. In each permutation, a PLS-DA model was built between the data and the permuted class labels using the optimal number of components determined by cross-validation for the model based on the original class assignment [28]. Metabolite set enrichment analysis [29] was then performed by mapping the metabolite data on the Human Metabolome Database (HMDB) [30]. Significantly enriched metabolites were identified using the global test [31] and the “betweenness centrality” measure to estimate metabolite importance followed by an assessment of pathway importance of each identified metabolite [32]. *P* values were adjusted for multiple testing using the Holm-Bonferroni method [33].

Similar to our previous work, single metabolite associations were evaluated using multivariable logistic regression models [20]. Specifically, for each metabolite we performed logistic regression with 25(OH)D >15 ng/ml as the outcome, after adjustment for age, gender, race, malignancy status, sepsis, and renal function (as estimated by glomerular filtration rate-modification of diet in renal disease (GFR-MDRD)). Additionally, for each metabolite we performed logistic regression with 28-day mortality as the outcome, after adjustment for Acute Physiology and Chronic Health Evaluation II (APACHE II) scores. Analyses were performed using STATA 14.1MP (College Station, TX, USA). A network of protein-protein and metabolite-protein interactions was then generated using the Search Tool for Interactions of Chemicals (STITCH) database, version 4.0 [34, 35]. STITCH active prediction methods are based on neighborhood, gene fusion, co-occurrence, co-expression, experiments, databases, text mining, and predictions, with a required confidence threshold (score) of 0.40 [34, 35].

## Results

Table 1 shows the demographic characteristics of the study cohort. Most patients were male (58%) and white (83%). The mean (SD) age at ICU admission was 55 (15) years. The mean (SD) 25(OH)D concentration was 20 (16) ng/ml, and 63% of cohort patients were diagnosed with sepsis. The mean APACHE II score was 26 (10). The 28-day mortality within the cohort was 35%. There were no significant differences between patients with 25(OH)D ≤ 15 ng/ml relative to those 25(OH)D > 15 ng/ml regarding any key baseline characteristic or for 28-day mortality.

**Table 1:** Cohort characteristics stratified by vitamin D status

	25(OH)D ≤15 ng/ml N = 24	25(OH)D >15 ng/ml N = 41	Total N = 65	*P* value
Age years, mean *±* SD	54.6 ± 13.7	55.8 ± 16.2	55.3 ± 15.2	0.77
Male gender, *N* (%)	16 (67)	22 (54)	38 (58)	0.30
White race, *N* (%)	19 (79)	35 (85)	54 (83)	0.71
APACHE II, mean *±* SD	26.7 ± 8.4	25.0 ± 10.4	25.6 ± 9.7	0.52
Peak creatinine*,* mean ± SD	2.7 ± 2.8	1.9 ± 1.5	2.2 ± 2.1	0.15
Malnutrition, *N* (%)	9 (38)	16 (39)	25 (38)	0.90
Malignancy, *N* (%)	10 (42)	15 (37)	25 (38)	0.68
Glomerular filtration rate, mean *±* SD	62.7 ± 49.9	57.8 ± 39.2	59.6 ± 43.1	0.66
Sepsis, *N* (%)	13 (54)	28 (68)	41 (63)	0.26
Sepsis with ARDS, *N* (%)	8 (33)	15 (37)	23 (35)	0.63
Sepsis without ARDS, *N* (%)	5 (21)	13 (32)	18 (28)	0.63

*APACHE II* Acute Physiology and Chronic Health Evaluation, *ARDS* acute respiratory distress syndrome. Plasma vitamin D deficiency is defined as 25(OH)D ≤ 15 ng/ml and vitamin D sufficiency is defined as 25(OH)D > 15 ng/ml

### Primary outcome

Metabolomic profiles differed in critically ill patients with 25(OH)D ≤ 15 ng/ml relative to those with levels >15 ng/ml. The supervised PLS-DA showed that the two different groups were well-clustered, with specific metabolic profiles for each (Fig. 2). Group membership (25(OH)D ≤ 15 ng/ml vs. >15 ng/ml) is illustrated by the 95% confidence ellipses calculated from PLS-DA scores. The permutation test with a *p* value of 0.033 indicates that the classification of global metabolite profiles by 25(OH)D is significantly different.

We utilized the random forest (RF) learning algorithm to select relevant variables for vitamin D status classification by estimating the importance of each metabolite to vitamin D status. In the RF analysis the “mean decrease accuracy” indicates how much a certain metabolite contributes to separation of the 25(OH)D groups, and the overall “predictive accuracy” is indicative of the accuracy for a set of metabolites to discriminate vitamin D status [32]. RF analysis of blood-targeted metabolomics data defined a set of 15 metabolites that constitute the best predictors of vitamin D status (Fig. 3). In particular, increased 1,5-anhydroglucitol, tryptophan betaine and 3-hydroxyoctanoate and decreased 2-arachidonoylglycerophosphocholine and N-6-trimethyllysine were strong predictors of 25(OH)D >15 ng/mL. These metabolites are products of carbohydrate, amino acid, lipid, lipid and amino acid metabolism, respectively. We found that in logistic regression, the combination of these 5 metabolites produced an area under the curve (AUC) for discrimination for 25(OH)D > 15 ng/ml of 0.82 (95% CI 0.71–0.93).

We next sought to identify differential biologically meaningful metabolite pathways in the cohort with regard to vitamin D status. Metabolite set enrichment analysis identified metabolites that were significantly enriched in patients with 25(OH)D > 15 ng/ml, with the strongest enrichment identified for glutathione metabolism (*p* = 0.020) and glutamate metabolism (*p* = 0.039). The metabolite sets related to glutathione metabolism (inclusive of cysteinylglycine, pyroglutamine, and L-cysteine) and glutamate metabolism (inclusive of glutamate and α-ketoglutarate) were enriched with regard to vitamin D status more than expected by chance (Additional files 2 and 3).

**Fig. 4:**
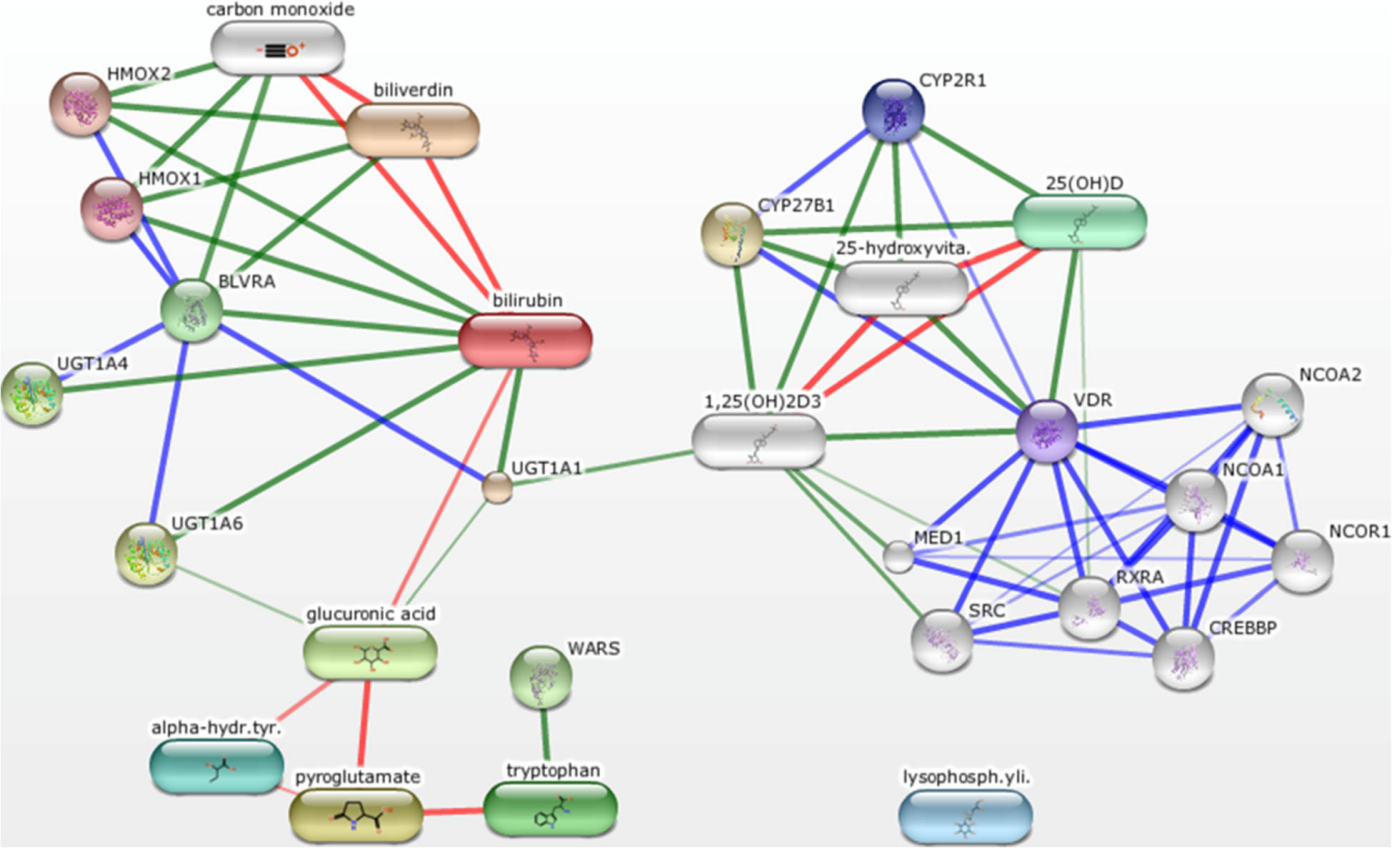
Network of metabolite-protein interactions inferred by metabolomics analysis. The names of the seven metabolites associated with vitamin D status and 28-day mortality (listed in Table 2), in addition to “vitamin D”, were used as input to generate a network of protein-protein and metabolite-protein interactions using the Search Tool for Interactions of Chemicals (STITCH) database. Network nodes are represented as either *cylinders* (chemicals) or *circles* (proteins, i.e. predicted functional partners), where nodes are *colored* if they are directly linked to the input, or *white* if they are of a higher iteration/depth (i.e. inferred by the network). *Lines* between nodes (edges) indicate predicted functional links, where stronger associations are represented by *thicker lines*; protein-protein interactions are shown in *blue*, chemical-protein interactions are shown in *green*, and interactions between chemicals are shown in *red*. Links between chemicals are not used to extend the network

Twenty metabolites were associated with vitamin D status at a nominal significance level (*p* < 0.05) in the RoCI cohort, after adjusting for age, race, malignancy status, sepsis, and renal function (Table 2). Seven of these metabolites were associated with vitamin D status and 28-day mortality: (1) glucuronate; (2) 1-palmitoyl-glycerophosphoinositol; (3) bilirubin (E,E) isomer; (4) pyroglutamine; (5) 2-hydroxybutyrate; (6) biliverdin; and (7) tryptophan (Table 2, Additional file 4). Network modeling of chemical-protein interactions was then utilized to illustrate the importance of the relationship between 25(OH)D and the metabolism of bilirubin, fatty acid derivatives, and bile acids through glucuronidation (Fig. 4).

**Table 2:** Top 20 associated metabolites by logistic regression analysis

Metabolite	Odds ratio adjusted vitamin D sufficiency^a^	P	Odds ratio adjusted 28-day mortality^b^	*P* value	Class
1,5-Anhydroglucitol	2.92	0.001	0.97	0.85	Carbohydrate
Methylglutaroylcarnitine	0.31	0.002	1.07	0.70	Amino acid
***Glucuronate***	0.52	0.005	1.54	0.018	Carbohydrate
2-Hydroxyisobutyrate	0.52	0.011	1.32	0.20	Amino acid
***1-Palmitoylglycerophosphoinositol***	2.36	0.018	1.95	0.028	Lipid
4-Methyl-2-oxopentanoate	2.77	0.019	1.20	0.63	Amino acid
C-glycosyltryptophan	0.34	0.020	1.57	0.14	Amino acid
***Bilirubin (E,E) isomer***	2.18	0.021	2.36	***0.005***	Cofactors
***Pyroglutamine***	1.99	0.023	2.51	***0.004***	Amino acid
Tryptophan betaine	1.60	0.026	1.03	0.86	Amino acid
4-Acetamidobutanoate	0.50	0.027	1.36	0.20	Amino acid
3-Hydroxyoctanoate	2.46	0.030	1.55	0.17	Lipid
Prolylhydroxyproline	0.53	0.036	1.27	0.30	Peptide
Pseudouridine	0.38	0.038	1.55	0.17	Nucleotide
N-acetylalanine	0.23	0.045	2.53	0.081	Amino acid
***2-Hydroxybutyrate***	1.90	0.046	1.84	0.044	Amino acid
***Biliverdin***	1.72	0.048	2.34	***0.003***	Cofactors
N-acetylneuraminate	0.49	0.048	1.38	0.24	Carbohydrate
***Tryptophan***	2.39	0.049	2.83	0.031	Amino acid
4-Androsten-3beta,17beta-diol disulfate 1	0.66	0.050	1.09	0.57	Lipid

Metabolite levels were log-transformed for analysis. The seven metabolites significantly associated with vitamin D status and 28-day mortality are shown in italic text under “Metabolite”. Odds ratios <1.00 indicate association between a metabolite and 25(OH)D ≤ 15 ng/ml. The significance threshold was set at *p* < 0.05

^a^Odds ratios and *p* values are for association with plasma vitamin D sufficiency (25(OH)D > 15 ng/ml), after adjustment for age, gender, race, sepsis, glomerular filtration rate and malignancy status

^b^Odds ratios and *p* values are for association with 28-day mortality after adjustment for Acute Physiology and Chronic Health Evaluation II

## Discussion

In the present study, our goal was to investigate whether vitamin D status in the early course of severe critical illness was associated with differences in the metabolic profiles of critically ill patients. Utilizing several analytic strategies, we demonstrated that the metabolic profile of critically ill patients differs based on their vitamin D status and there is evidence that metabolites related to vitamin D status are most prominently related to glutathione and glutamate metabolism and glucuronidation.

In humans, 25(OH)D is the major circulating form of vitamin D_3_. Steady-state plasma 25(OH)D concentrations represent a balance between formation and clearance activities, which are mediated by phase I and phase II oxidation and conjugation processes. Variation in the efficiency of these detoxification reactions contributes to variability in circulating plasma concentrations of 25(OH)D, thereby altering the activity of this prohormone. Based on the results of this metabolomic profiling study, we have identified two important phase II metabolism pathways for glutamate, glucuronidation and glutathione cycling that are associated with vitamin D homeostasis in critically ill ICU patients.

By MSEA, we identified the overlapping pathways for glutathione and glutamate metabolism as the most highly enriched pathways in our metabolite data. Pyroglutamine, a cyclic metabolite of glutamine and component of the glutathione cycle, was identified in PLS-DA analysis as a classifier of vitamin D status (Fig. 3). Glutathione, a major cellular thiol antioxidant, is a cofactor of the enzymatic detoxification of oxygen radicals [36, 37]. In vitro data suggest that vitamin D upregulates cellular glutathione [36]. Furthermore, in community-dwelling adults, serum 25(OH)D levels are associated with increased circulating glutathione [38]. Though the redox state of reduced/oxidized glutathione (GSH/GSSG) is closely regulated, it decreases with tissue injury, inflammation, sepsis, and toxin exposure [39–41]. Oxidative stress is well-described in patients with sepsis, with supporting evidence for production of reactive oxygen species (ROS) and associated damage [42]. In patients with sepsis, inflammatory response initiation via oxidative stress occurs through redox pathway activation of nuclear factor κB (NFκB) and expression of a substantial number of genes involved in the immune response and cell survival [43, 44].

Glutamate, a highly concentrated intracellular amino acid is important for biosynthesis of multiple amino acids, nucleic acids, nucleotides and metabolites [45]. Though glutamate has a low concentration in plasma [46] it has an important role in peripheral organs and tissues as an extracellular signal mediator [47]. More germane to the severely ill cohort under study, ionotropic glutamate receptors are expressed on T cells and B cells. Dendritic cells and macrophages and glutamate serve as an immunomodulator in the initiation and development of T-cell-mediated immunity in peripheral tissues [48, 49]: α-ketoglutarate, a Krebs cycle intermediate, is produced in a glutamine-dependent fashion and regulates the T helper 1 cell and regulatory T cell generation balance [50].

Glucuronidation is crucial for the hepatic and renal metabolism of compounds, including bile acids, steroids, bilirubin, and fatty acids, to facilitate their elimination from the body and to improve the disposition and activity of drugs and hormones across tissues. Glucuronidation is an essential chemical reaction for rendering 1, 25(OH)_2_D_3_ (the most metabolically active vitamin D metabolite) to a water-soluble, biologically inactive form, but may also serve as a reservoir for enterohepatic circulation [51]. This conjugation reaction is performed by UDP glucuronosyltransferase (UGT) enzymes in the liver. In addition to glucuronate, we also identified bilirubin and biliverdin as important metabolite predictors of vitamin D status. Bilirubin, a metabolite of the heme end product biliverdin, is glucuronidated by UGT1A isoforms, chiefly UGT1A1, in addition to UGT1A4.

While UGTs catalyze the conjugation of a wide variety of endogenous substrates, recent studies have identified UGT1A4 as the primary catalyst of 25(OH)D glucuronidation in vivo [52]. Failure to recycle glucuronides could contribute to low vitamin D status through promoting the metabolism of 25(OH)D to its inactive, polar forms, which are more readily excreted, thereby reducing its levels in the systemic circulation. In addition, as UGT enzymes are highly polymorphic, and “gain-of-function” variants with high substrate clearance activity have been described in humans [52, 53], inter-individual variation in 25(OH)D levels due to variable UGT1A4 activity could contribute to lower 25(OH)D levels in circulation. Because homozygous carriers of UGT1A4*3 demonstrate enhanced 25(OH)D glucuronidation activity, patients with this genotype might be expected to have lower circulating levels of 25(OH)D and may therefore be at greater risk of low vitamin D status [52, 53]. In addition to UGT1A4, UGT1A1 was also predicted by network modeling of chemical-protein interactions for all seven metabolites, in addition to vitamin D metabolites, to co-interact with 1,25(OH)_2_D_3_, bilirubin, and glucuronic acid (Fig. 4). A specific role for UGT1A1 in glucuronidation of vitamin D has not been investigated but may contribute to vitamin D status in critically ill patients.

Two additional metabolites, 1-palmitoyl-glycerophosphoinositol and 2-hydroxybutyrate, were also associated with 25(OH)D plasma levels (Table 2). While little is known about the specific roles of 1-palmitoyl-glycerophosphoinositol in vitamin D metabolism, this compound belongs to the glycerophosphoinositol family and, along with pyroglutamine and 2-hydroxyisobutyrate (a derivative of 2-hydroxybutyrate), was associated with anti-hypertensive and lipid-lowering drugs in serum samples from a study of 1762 participants in the Cooperative Health Research in the Region of Augsburg (KORA) study [54].

The present study is not without potential limitations. Metabolites were measured early in the ICU course of severe critical illness, from a relatively small number of patients, at a single time point, and from a single biofluid (plasma). As the timing of plasma collection was within 72 hours of ICU admission and not at a uniform time point, the potential for variability and switches in metabolic pathways during the course of critical illness cannot be excluded. Our observational study included patients who were critically ill for various reasons, creating a heterogeneous study sample with high severity of illness. Further, selection bias may be present as we are analyzing only a subset of patients in the RoCI cohort. Without a control population of healthy vitamin-D-sufficient individuals, we do not have comparative metabolomic information on vitamin D status in the critically ill relative to the control. We are unable to account for the impact of race on metabolic profiles as our cohort was mostly white. Though we do have information on nutrition status, we do not have information related to nutrition intake, body mass index (BMI) or alcohol intake at the time metabolomic profiles were obtained. As our study was performed on a convenience sample, our results may not be generalizable to all critical care patients. Our bioinformatics approaches, while robust, are not without risk of introducing sources of bias. Although PLS-DA is well-suited for metabolomic data with much larger numbers of predictors than observations and multi-collinearity [55], it may be subject to over-fitting; to limit this, we performed cross-validation and permutation testing [56, 57]. Our measurement of 25(OH)D in a critically ill population with a mean estimated GFR of 59.6 ml/minute may not accurately account for the biologically active form of vitamin D. Our data do not allow for the distinction between metabolites that may be on a causal pathway or simply confounders of the association between vitamin D and outcome. Further, though aging is noted to be an important factor in metabolic homeostasis [25, 58] our study age range cannot account for such alterations. Finally, we cannot fully account for potential confounding, reverse causation, and the lack of a randomly-distributed exposure [59].

## Conclusion

In summary, vitamin D status is associated with differential metabolic profiles in early severe critical illness. Glutathione and glutamate metabolism, which play principal roles in redox regulation and immunomodulation, respectively, were significantly altered with vitamin D status.

## Additional files

Additional file 1: Supplemental methods. (DOC 100 kb)

Additional file 2: Figure S1. Glutathione pathway metabolite member normalized concentrations from pathway analysis by global metabolomics in plasma from 24 patients with 25(OH)D ≤15 ng/ml (*red*) and 41 patients with 25(OH)D >15 ng/ml (*green*). (PPTX 53 kb)

Additional file 3: Figure S2. Glutamate metabolism pathway metabolite member normalized concentrations from pathway analysis by global metabolomics in plasma from 24 patients with 25(OH)D ≤15 ng/ml (*red*) and 41 patients with 25(OH)D >15 ng/ml (*green*). (PPTX 46 kb)

Additional file 4: Table S1. Mortality and 25(OH)D associated metabolites by logistic regression analysis. (DOC 35 kb)

## Abbreviations

APACHE II: Acute Physiology and Chronic Health Evaluation II
ARDS: Acute respiratory distress syndrome
AUC: Area under the curve
GFR: Glomerular filtration rate
ICU: Intensive care unit
IL: Interleukin
PLS-DA: Partial least squares-discriminant analysis
RF: Random forest
RoCI: Registry of Critical Illness
SIRS: Systemic inflammatory response syndrome
STITCH: Search Tool for Interactions of Chemicals
UGT: Glucuronosyltransferase
