# Supplementary material for: Metabolome alterations in severe critical illness and vitamin D status
Source: Crit Care. 2017 Jul 28;21:193. doi: 10.1186/s13054-017-1794-y (PMC5532782; doi:10.1186/s13054-017-1794-y)
Supplement: Supplementary file 1 — Supplemental methods. (DOC 100 kb) [file 13054_2017_1794_MOESM1_ESM.doc]

**Supplemental Methods**

The Brigham and Women’s Hospital Registry of Critical Illness (RoCI) is approved by the Partners IRB committee (2008-P-000495). The protocol for recruitment for RoCI has been published in detail elsewhere (1). Briefly, adult patient (over age 18) who is admitted to the BWH Medical Intensive Care Unit is eligible for inclusion in the RoCI within 72 hours of presentation, unless certain exclusion criteria are met (unable to provide consent due to cognitive dysfunction or no appropriate health care proxy, prior refusal, admission purely for comfort care, Jehova’s Witness status, or a baseline hemoglobin <8 g/dL or hemoglobin <9 g/dL with either admission for active bleeding or with acute ischemia). Plasma is obtained on Days 1, 3, and 7 of enrollment. Extensive phenotypic data (including age, gender, key comorbidities, and APACHE II score), laboratory and radiologic data are recorded for all subjects. Classification of Systemic Inflammatory Response (SIRS), Sepsis, and ARDS is determined by a consensus panel of ICU physicians using the current disease classification (2, 3).

We utilized metabolomic data that was produced by Rogers et.al. (4) from the Registry of Critical Illness (RoCI) cohort described by Dolinay et.al. (1). Among 225 subjects described by Dolinay et.al. (1), 90 subjects were selected for metabolic profiling by Rogers et.al.: 29 with SIRS, 30 with Sepsis, and 31 with sepsis-induced ARDS (4). Cases for the Rogers paper were selected for metabolic profiling based in part on IL-18 levels as part of the Dolinay et.al. study protocol (sepsis and SIRS patients with low IL-18 levels, ARDS with high IL-18 levels) (1). Rogers published metabolomic signatures of outcome in the RoCI and a second cohort (4). Of the 90 RoCI subjects with metabolite profiles completed in the Rogers study, 65 had enough plasma available to determine 25(OH)D. We studied these 65 patients: 24 with SIRS, 21 with Sepsis, and 20 with sepsis-induced ARDS (Table 1).

For most of the cohort patients, the blood samples were obtained very early in hospitalization. 51 patients were admitted to the ICU within 24 hours of hospital admission. 6 patients were admitted within 24 hours and 7 days of hospital admission. 8 patients were admitted to the ICU after 7 days of hospital admission.

Blood samples were drawn and transferred into EDTA coated blood collection tubes within 24 hours from study inclusion and processed within 4 hours after venipuncture. Subsequently, plasma was fractionated, aliquoted and stored at -80°C (1). 150 μl aliquots were shipped on dry-ice to Metabolon, Inc. Following receipt, the frozen samples in the box were immediately stored at -80^o^C. Metabolites are noted by other investigators to be stable for at least two freeze thaw cycles (5).

The exposure of interest was serum 25(OH)D and categorized *a priori* as deficiency (25(OH)D ≤15 ng/mL). Although the Institute of Medicine report (6) and the Endocrine Society Clinical Practice Guideline (7) suggested 25(OH)D < 20 ng/ml as the cut-off point for deficiency, large studies performed by our group have consistently found in our ICU population under study that outcomes are associated with 25(OH)D ≤ 15 ng/mL (8-12).

All patients in the analytic cohort had at least 28-day follow up data available for review. 28 day mortality was determined on the complete cohort using hospital records and the Social Security Administration Death Master File (13). Race was either self-determined or designated by a patient representative/healthcare proxy. Presence of malignancy was determined by subject interview and medical record confirmation. Renal function, measured by glomerular filtration rate (GFR) was calculated with the Modification of Diet in Renal Disease (MDRD) equation from the baseline creatinine, age, gender, and race of cohort patients (33)*.* Classification of SIRS, sepsis, and ARDS, was determined by a consensus panel of ICU physicians using the Consensus Conference classifications (2, 3). Acute physiology and chronic health evaluation (APACHE) II score was determined at 24 hours post-ICU admission (14). Malnutrition was considered to be present if the patient was diagnosed by a Registered Dietitian, within 10 days prior to or 2 days after ICU admission, to have either nonspecific protein/caloric malnutrition or specific (mild, moderate or severe) protein/caloric malnutrition (15, 16).

For the metabolite measurements, instrument variability was determined by calculating the median relative standard deviation (RSD) for the internal standards that were added to each sample prior to injection into the mass spectrometers. The median relative standard deviation for instrument variability was 5%. Overall process variability was determined by calculating the median RSD for all endogenous metabolites (i.e., non-instrument standards) present in 100% of the Client Matrix samples, which are technical replicates of pooled client samples. The median relative standard deviation for overall process variability was 11% (4).

**Supplemental Methods References**

1. Dolinay T, Kim YS, Howrylak J, Hunninghake GM, An CH, Fredenburgh L, Massaro AF, Rogers A, Gazourian L, Nakahira K, Haspel JA, Landazury R, Eppanapally S, Christie JD, Meyer NJ, Ware LB, Christiani DC, Ryter SW, Baron RM, Choi AM. Inflammasome-regulated cytokines are critical mediators of acute lung injury. Am J Respir Crit Care Med 2012;185(11):1225-1234.

2. Levy MM, Fink MP, Marshall JC, Abraham E, Angus D, Cook D, Cohen J, Opal SM, Vincent JL, Ramsay G. 2001 SCCM/ESICM/ACCP/ATS/SIS International Sepsis Definitions Conference. Crit Care Med 2003;31(4):1250-1256.

3. Bernard GR, Artigas A, Brigham KL, Carlet J, Falke K, Hudson L, Lamy M, Legall JR, Morris A, Spragg R. The American-European Consensus Conference on ARDS. Definitions, mechanisms, relevant outcomes, and clinical trial coordination. Am J Respir Crit Care Med 1994;149(3 Pt 1):818-824.

4. Rogers AJ, McGeachie M, Baron RM, Gazourian L, Haspel JA, Nakahira K, Fredenburgh LE, Hunninghake GM, Raby BA, Matthay MA, Otero RM, Fowler VG, Rivers EP, Woods CW, Kingsmore S, Langley RJ, Choi AM. Metabolomic derangements are associated with mortality in critically ill adult patients. PLoS One 2014;9(1):e87538.

5. Breier M, Wahl S, Prehn C, Fugmann M, Ferrari U, Weise M, Banning F, Seissler J, Grallert H, Adamski J, Lechner A. Targeted metabolomics identifies reliable and stable metabolites in human serum and plasma samples. PLoS One 2014;9(2):e89728.

6. Ross AC, Manson JE, Abrams SA, Aloia JF, Brannon PM, Clinton SK, Durazo-Arvizu RA, Gallagher JC, Gallo RL, Jones G, Kovacs CS, Mayne ST, Rosen CJ, Shapses SA. The 2011 report on dietary reference intakes for calcium and vitamin D from the Institute of Medicine: what clinicians need to know. J Clin Endocrinol Metab 2011;96(1):53-58.

7. Holick MF, Binkley NC, Bischoff-Ferrari HA, Gordon CM, Hanley DA, Heaney RP, Murad MH, Weaver CM. Evaluation, treatment, and prevention of vitamin D deficiency: an Endocrine Society clinical practice guideline. J Clin Endocrinol Metab 2011;96(7):1911-1930.

8. Braun A, Chang D, Mahadevappa K, Gibbons FK, Liu Y, Giovannucci E, Christopher KB. Association of low serum 25-hydroxyvitamin D levels and mortality in the critically ill. Crit Care Med 2011;39(4):671-677.

9. Braun AB, Gibbons FK, Litonjua AA, Giovannucci E, Christopher KB. Low serum 25-hydroxyvitamin D at critical care initiation is associated with increased mortality. Crit Care Med 2012;40(1):63-72.

10. Braun AB, Litonjua AA, Moromizato T, Gibbons FK, Giovannucci E, Christopher KB. Association of low serum 25-hydroxyvitamin D levels and acute kidney injury in the critically ill*. Crit Care Med 2012;40(12):3170-3179.

11. Moromizato T, Litonjua AA, Braun AB, Gibbons FK, Giovannucci E, Christopher KB. Association of low serum 25-hydroxyvitamin D levels and sepsis in the critically ill. Crit Care Med 2014;42(1):97-107.

12. Thickett DR, Moromizato T, Litonjua AA, Amrein K, Quraishi SA, Lee-Sarwar KA, Mogensen KM, Purtle SW, Gibbons FK, Camargo Jr CA, Giovannucci E, Christopher KB. Association between pre-hospital vitamin D status and incident acute respiratory failure in critically ill patients: a retrospective cohort study. BMJ Open Respiratory Research 2015;2:e000074.

13. Sohn MW, Arnold N, Maynard C, Hynes DM. Accuracy and completeness of mortality data in the Department of Veterans Affairs. Popul Health Metr 2006;4:2.

14. Knaus WA, Draper EA, Wagner DP, Zimmerman JE. APACHE II: a severity of disease classification system. Crit Care Med 1985;13(10):818-829.

15. Robinson MK, Mogensen KM, Casey JD, McKane CK, Moromizato T, Rawn JD, Christopher KB. The relationship among obesity, nutritional status, and mortality in the critically ill*. Crit Care Med 2015;43(1):87-100.

16. Mogensen KM, Robinson MK, Casey JD, Gunasekera NS, Moromizato T, Rawn JD, Christopher KB. Nutritional Status and Mortality in the Critically Ill*. Critical care medicine 2015;43(12):2605-2615.
