# Supplementary figures and images for: Metabolome alterations in severe critical illness and vitamin D status
Source: Crit Care. 2017 Jul 28;21:193. doi: 10.1186/s13054-017-1794-y (PMC5532782; doi:10.1186/s13054-017-1794-y)

## Slide 1
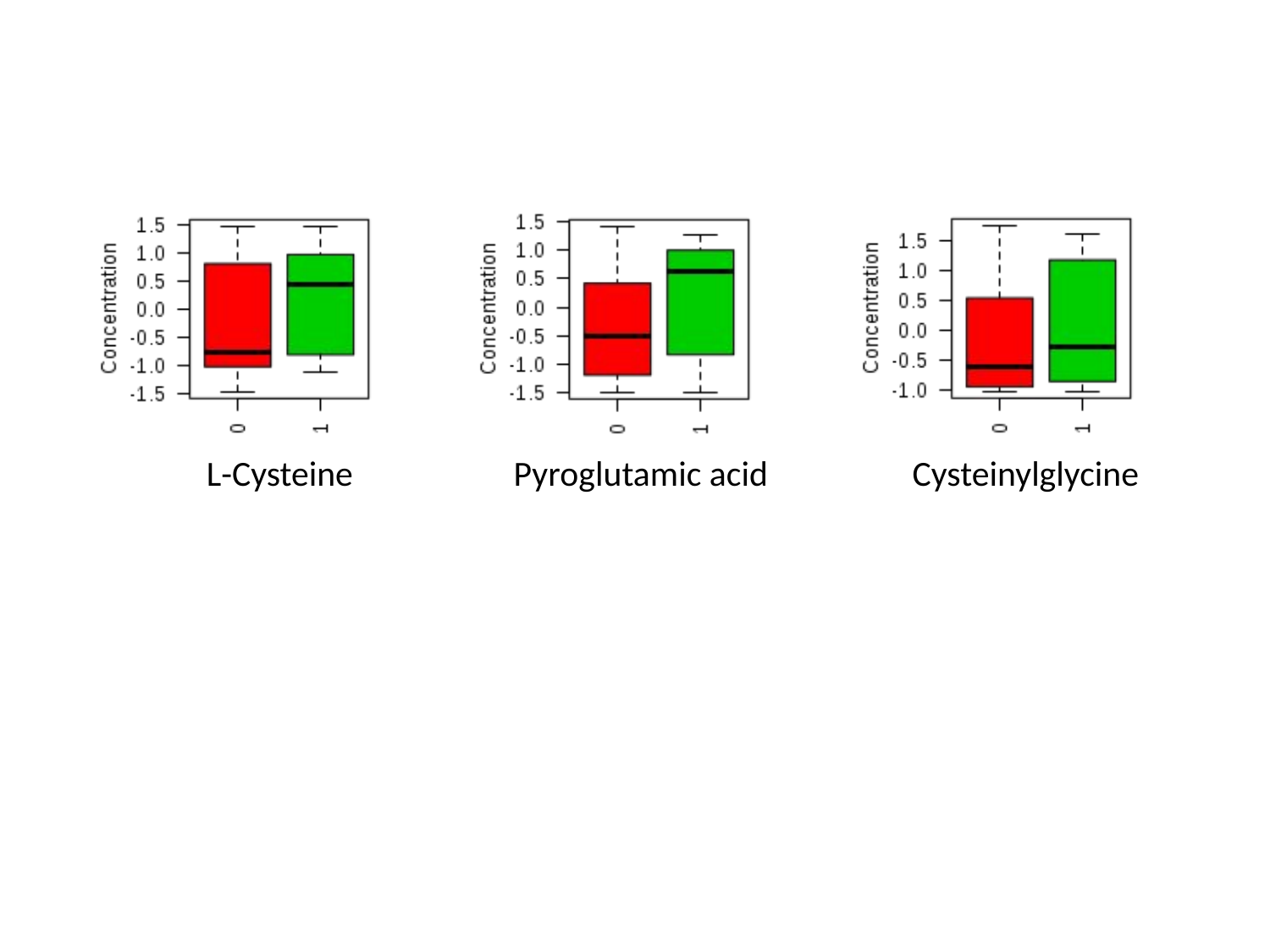

L-Cysteine Pyroglutamic acid Cysteinylglycine

Supplement: Supplementary file 2 — Glutathione pathway metabolite member normalized concentrations from pathway analysis by global metabolomics in plasma from 24 patients with 25(OH)D ≤15 ng/ml (red) and 41 patients with 25(OH)D >15 ng/ml (green). (PPTX 53 kb) [file 13054_2017_1794_MOESM2_ESM.pptx]

## Slide 1
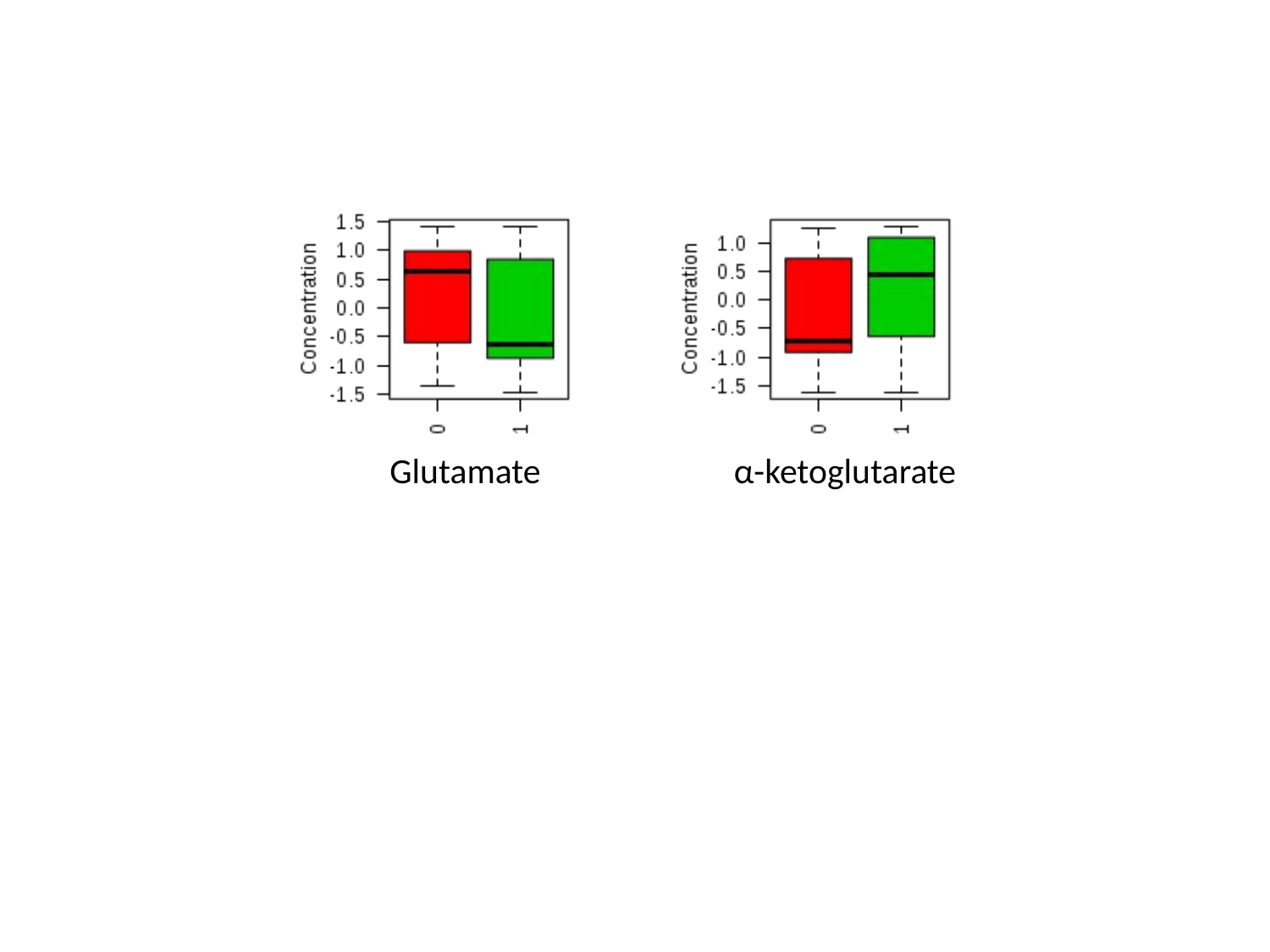

Glutamate	 α-ketoglutarate

Supplement: Supplementary file 3 — Glutamate metabolism pathway metabolite member normalized concentrations from pathway analysis by global metabolomics in plasma from 24 patients with 25(OH)D ≤15 ng/ml (red) and 41 patients with 25(OH)D >15 ng/ml (green). (PPTX 46 kb) [file 13054_2017_1794_MOESM3_ESM.pptx]
