# Supplementary material for: Metabolome alterations in severe critical illness and vitamin D status
Source: Crit Care. 2017 Jul 28;21:193. doi: 10.1186/s13054-017-1794-y (PMC5532782; doi:10.1186/s13054-017-1794-y)
Supplement: Supplementary file 4 — Mortality and 25(OH)D associated metabolites by logistic regression analysis. (DOC 35 kb) [file 13054_2017_1794_MOESM4_ESM.doc]

**Table S1. Mortality and 25(OH)D** associated metabolites by logistic regression analysis.

| Metabolite | **Odds Ratio^c^** | **P^c^** | **Odds Ratio^d^** | **P^d^** | **Class** |
| --- | --- | --- | --- | --- | --- |
| **glucuronate** | 1.54 | 0.018 | 1.72 | 0.008 | Carbohydrate |
| **1-palmitoylglycerophosphoinositol** | 1.95 | 0.028 | 1.91 | 0.043 | Lipid |
| **bilirubin (E,E) isomer** | 2.36 | 0.005 | 2.33 | 0.006 | Cofactors |
| **pyroglutamine** | 2.51 | 0.004 | 2.49 | 0.006 | Amino acid |
| **2-hydroxybutyrate** | 1.84 | 0.044 | 1.79 | 0.060 | Amino acid |
| **biliverdin** | 2.34 | 0.003 | 2.30 | 0.003 | Cofactors |

Metabolite levels were log-transformed for analysis. The seven metabolites significantly associated with vitamin D status and 28 day mortality are shown in bolded text in column 1.

**Note:** β and P values shown are for results using logistic regression for association with

**c**. 28 day mortality after adjustment for APACHE II.

d. 28 day mortality after adjustment for APACHE II and vitamin D sufficiency (25(OH)D3>15ng/ml).

Significance threshold is P<0.05.
